# Perennial and annual cropping sequences differentially influence soil functionality in Luvisolic soils

**DOI:** 10.1038/s41598-026-59597-2

**Published:** 2026-08-18

**Authors:** Keshav Bhattarai, Nityananda Khanal, Malinda S. Thilakarathna, Newton Z. Lupwayi, Noabur Rahman, Bharat M. Shrestha, Reynald L. Lemke, Roland Kroebel

**Affiliations:** 1 Agriculture and Agri-Food Canada, Beaverlodge Research Farm, Beaverlodge, AB T0H 0C0 Canada; 2 Department of Agricultural, Food and Nutritional Science, University of Alberta, Edmonton, AB T6G 2P5 Canada; 3 Agriculture and Agri-Food Canada, Lethbridge Research and Development Centre, Lethbridge, AB T1J 4B1 Canada; 4 Agriculture and Agri-Food Canada, Saskatoon Research and Development Centre, Saskatoon, SK S7N 5E2 Canada

**Keywords:** Ecology, Ecology, Environmental sciences, Plant sciences

## Abstract

Diversified cropping systems are vital for improving soil properties and resilience. This study evaluated the impact of integrating perennial forage crops into annual systems under varying nitrogen (N) levels on a Luvisolic soil, utilizing 10-year experimental plots (2013–2023) in the Peace region of northwestern Canada. The experiment in a split-plot design with four replicates had eight cropping sequences as main plots and three N rates (0, 45, and 90 kg N ha^− 1^) as sub-plots. Soil samples from 0 to 15 cm depths collected in 2023 were analyzed for physical, chemical, and biological properties, and data were assessed using linear mixed models and principal component analysis (PCA). Crop diversification, including legumes and perennials improved soil aggregate stability, water-stable macro-aggregates, and enzyme activities, while N fertilization had no measurable long-term effects on these parameters. The turfgrass to legume sequence (S_7_) and the annual to perennial integrated sequence (S_3_) increased mean weight diameter (MWD) by 87% and 29%, respectively, relative to the conventional annual rotation. The diversified, fescue-integrated cropping sequences clearly excelled in soil structural stability parameters, increasing water-stable macroaggregates by up to 57%. PCA aligned carbon pools, structural stability, and microbial activity along with the major axis, indicating that these soil attributes collectively explained much of the observed functional variation and were more strongly influenced by crop functional group than by individual cropping sequences. Moreover, most cropping sequences exhibited overlapping soil property profiles, indicating that responses were not uniform across all systems. Integrating legumes and perennial crops into annual systems can contribute to improved soil structural stability, and biological functioning, thereby supporting sustainable cropping in the Peace region of Canada.

**Supplementary Information:**

The online version contains supplementary material available at 10.1038/s41598-026-59597-2.

## Introduction

Extensive research over the past several decades has established diversified cropping as a fundamental agroecological principle for enhancing soil health, improving nutrient cycling, leveraging natural pest regulation, and increasing primary productivity^1^. Crop diversification, together with the zero-tillage, became the most prominent and widely adopted production practices across the farming systems^2^. Greater rotational diversity not only reduces weed, pest, and disease pressure, but also improves input use efficiency, enhances soil health, increases overall system productivity, ultimately strengthening the competitiveness of agricultural systems in global export markets^3–5^. However, cropping decisions depend on the availability of agronomic management options suited to operate local soil and climatic conditions, production costs, and market forces, which can constrain both ecological and economic potential^6^.

The inclusion of pulses (annual grain legumes) in rotations is widely recognized as a cornerstone practice for supporting nutrient cycling, enhancing organic carbon (SOC) and sustaining soil health^7,8^. Recent studies further highlight that perennial forage legumes provide even greater long-term benefits for soil quality due to their extended growing periods, higher root-to-shoot ratio, and superior nitrogen-fixing capacity compared to annual pulse crops^9,10^. Actively growing perennial forages help stabilize soil with their extensive root systems, thus reducing the risk of soil erosion by wind and water^11,12^. In addition, the fibrous root systems, along with polymer organics within root exudates, can effectively bind soil particles to increase aggregate size and structural stability^13^. The deep-growing root systems also have the potential to enhance nutrient (nitrate, phosphate, and sulfate) utilization, thereby reducing the risk of leaching into groundwater^14,15^. Despite the removal of aboveground biomass during harvest, the high belowground carbon (C) input of perennial forages results in soil organic matter (SOM) buildup^16^. Increased SOM levels are associated with higher microbial activity and nutrient cycling^17^. The diverse species of perennial forages release a multitude of compounds in the root exudates, which support the abundance and diversity of the microbial community^18^. Upon decomposition, the vigorous and deep root system of perennial forages alters soil porosity, enhances aeration and water movement, and reduces soil compaction problems^19^. Therefore, an improved subsoil condition can be achieved by perennial forage crop cultivation.

Climate change and increasing weather variability pose a significant challenge to agricultural production worldwide. In the Peace River region of western Canada, erratic weather patterns and more frequent extremes such as temperature fluctuations, droughts, and heavy precipitation, have heightened the vulnerability of agricultural productivity and ecosystem health^20,21^. Under these constraints and emerging uncertainties, innovations in crop diversification are essential for improving the resilience and sustainability of production systems. This study hypothesizes that integrating perennial forage seed crops into conventional annual cropping systems can deliver multiple benefits including greater system productivity, improved nitrogen use efficiency (NUE), increased SOM, enhance soil structure, stimulation of soil microbial activity, and augmentation of key enzymatic functions. Evaluation of eight cropping sequences under different N fertilizer levels (0, 45 and 90 kg N ha^− 1^) enabled assessment of the soil’s inherent N-supplying capacity and the role of crop diversification in reducing dependence on synthetic N inputs. Although N fertilization plays a vital role in increasing crop productivity, its effects on soil quality improvement are complex and often inconsistent. Usually, excessive or imbalanced N fertilization can accelerate SOM mineralization and reduce soil carbon stability^22^. On contrary, N application can influence soil functionality through their varying effects on root biomass, microbial activity, rhizodeposits, and enzymatic activities involved in carbon and nitrogen cycling depending on soil rhizosphere and cropping environments^23,24^. Therefore, the treatment design facilitated analysis of crop functional group interactions (annual/perennial, legume/nonlegume) under varying N availability to rationalize productivity, N economy, and soil functionality. While NUE and system profitability are addressed in a separate publication^25^, the present study examines the long-term impact of perennial-integrated crop diversification on soil quality, based on a decade of field experimentation from 2013 to 2023.

## Materials and methods

### Physiographic description of the study area

The study was performed in 2023 by using long-term experimental plots established in 2013 at the Agriculture and Agri-Food Canada’s Beaverlodge Research Farm (55°12’ N, 119°24’ W), Alberta, Canada. Detailed climatic and edaphic features of the study site are available in Bhattarai et al.^25^ and Khanal et al.^26^. Briefly, the soil is classified as a Hythe fine loam within the Dark Gray Luvisolic soil order^27^. Initial soil analyses indicated that the surface horizon is slightly acidic (pH = 5.4) with a loamy texture, consisting of 31.3% sand, 46.0% silt, and 22.7% clay, whereas the subsurface horizon is characterized as clay loam, comprising 28.1% sand, 36.1% silt, and 35.8% clay. The regional climate is cool continental, characterized by long, cold winters and short, warm summers, with moderate precipitation concentrated during the growing season from May to September. Based on climate records from 1995 to 2025 for the Beaverlodge area, the mean annual temperature was 2.6 °C, with mean monthly temperatures ranging from − 11.0 °C in January to 15.9 °C in July. Annual precipitation ranged from 262 to 586 mm, with a 30-year average of 391 mm.

### Experimental design and treatment details

The field experiment was set up in a split-plot design with four replicates. Experimental treatments comprised eight different cropping sequences that were assigned to main plots and three different N levels (0, 45, and 90 kg N ha^− 1^) constituted the sub-plot factors. Cropping sequence treatments comprised of the regionally most common annual crops (canola [*Brassica napus* L.], wheat [*Triticum aestivum* L.], pea [*Pisum sativum* L.], and barley [*Hordeum vulgare* L.]), perennial grasses (creeping red fescue [*Festuca rubra* L], meadow bromegrass [*Bromus riparius* Rehm], and timothy [*Phleum pratense* L]), and perennial forage legumes (alsike clover [*Trifolium hybridum* L.], and red clover [*Trifolium pratense* L.]). Cropping sequence treatments consisted of diversification options with varied combinations of species in sequence (Table 1). Similarly, three level of N supplementation were implemented to assess the soil’s inherent N supply (with 0 kg N ha^− 1^) and evaluate N economy in diversified cropping systems using partial (45 kg N ha^− 1^) and full rates (90 kg N ha^− 1^) based on soil test results, as reported by Khanal et al.^26^. This design also enables examination of crop functional group interactions arising from differences in N demand and contribution, thereby elucidating their effects on overall systems productivity and sustainability.

For the N treatments, urea fertilizer (46-0-0) was applied in bands for annual crops during seeding in the spring (May), whereas it was broadcasted over perennial forage crops in early fall (September). Other nutrients, including phosphorus, potassium, and sulfur were applied at 30, 20, and 20 kg ha^− 1^ of P_2_O_5_, K_2_O, and S, respectively during the seeding of each crop. The treatment factors were randomly assigned in the first year of experimentation and the layout was maintained throughout a long-term experimental period. Individual sub-plot size was 2.4 m × 8 m, where eight rows of crops were seeded at 30 cm spacing with a cross-slot seeder (Valmar Cross Slot IP Limited; Feilding, New Zealand). The experiment was conducted under a no-till management system from the year of establishment.

**Table 1:** Cropping sequences with its key features.

S.N.	Crops in sequences (2013-24)	Key/transitioning features
S_1_	W-C-W-C-W-C-W-C-FR-W-C-W	Conventional annual crop sequence
S_2_	P-B-W-C-P-W-C-P-FR-W-P-B	Diversified annual crop sequence
S_3_	C-C-C-C-P-MB-MB-MB-FR-P-RC/W-RC	Annual to perennial crop sequence
S_4_	RC-RC-W-C-RC/W-RC-W-C-FR-MB-MB-MB	Legume to vernalizing grass crop sequence
S_5_	AC-AC-W-C-AC/W-AC-W-C-FR-T-T-T	Legume to non-vernalizing grass crop sequence
S_6_	inRC-RC-W-C-RC/W-RC-C-W-FR-CF-CF-CF	Legume to turf grass crop sequence
S_7_	CF-CF-CF-C-P-CF-CF-CF-FR-P-AC/W-AC	Turf grass to legume crop sequence
S_8_	inAC-AC-W-C-AC/W-AC-CF-CF-FR-C-P-W	Diversified perennial to annual crop sequence

W, wheat; C, canola; FR, fall rye; P, peas; B, barley; MB, meadow bromegrass; RC, red clover; AC, alsike clover; T, timothy; inRC, red clover inoculated with *Rhizobium* inoculant; CF, creeping red fescue; inAC, alsike clover inoculated with *Rhizobium* inoculant. The dash sign ' - ' refers to the sequential crop and the slash sign ' / ' refers to the intercrop.

### Soil sampling and processing

Soil samples were collected from 0 to 15 cm depth in September 2023 (post-harvest) to analyze various physico-chemical properties across eight cropping sequences under three N fertilizer regimes. The samples were collected from each experimental unit using a Giddings soil corer (Giddings Machine Company, Windsor, CO). Visible root mass and plant debris were removed from the soil samples manually. The soil samples were then air-dried at room temperature, freed from coarse fragments (such as roots, gravels, etc.), ground using an electric grinder (Marathon Electric, Wausau, Wisconsin 54401), passed through a 2-mm sieve, and stored in plastic vials for physicochemical analyses in the laboratory.

For determining microbial parameters and extracellular enzyme activities (EEAs) soil samples were collected from 0 to 15 cm depth of each experimental unit using a hand auger during the early growing season in June. Those soil samples were transported immediately to the laboratory under cold conditions. After homogenization, sub-samples of freshly sieved (2-mm) soil were stored at 4 °C until further analyses.

### Soil analyses

Soil bulk density was determined with the post-harvest, undisturbed samples extracted using the core method^28^. Water content at field capacity was determined by saturating the soil with water through capillary rise, allowing it to drain for 48 h, and then measuring the water content using the gravimetric method^29^. Soil aggregate stability from each experimental unit was assessed using a wet-sieving apparatus by following the modified Yoder method^30^. Aggregates were categorized into macro- (1–4 mm), meso- (0.25–1 mm) and micro-aggregates (< 0.25 mm), and their relative proportion in the sample was expressed as a percentage of the total aggregates. For aggregate fractionation, 50 g of 4 mm sized air-dried soil was placed on the top sieve and pre-soaked with deionized water (22 °C) for 10 min. The assembly of sieves was then gently immersed in water for 10 min with a stroke length of 35 mm and a frequency of 16 cycles min^− 1^. The soil aggregates retained on each sieve were collected, oven-dried (50 °C) for 48 h, and then weighed for fraction measurements. The fraction < 0.125 mm was calculated from the difference between the initial soil weight and the sum of the other six size fractions. Soil aggregate stability MWD, which was calculated by summing the mean diameter of each size fraction multiplied by the fractional weight of each size fraction^30^.

Soil pH was measured using a pH meter (Accumet model AR50, Fisher Scientific Company, Ottawa, ON) on a solution obtained from a 1:2 soil-to-water ratio^31^. Soil total carbon (TC) and total nitrogen (TN) were determined at a 0–15 cm depth from post-harvest soil samples taken in 2023 using the dry combustion method^32^. For SOC, about 1 g of air-dried sample was first moistened with deionized water to its field capacity level and then fumigated with 12 M HCl in a desiccator^33^. After 24 h, samples were oven-dried at 50 °C using a ventilated drying oven to remove the residual moisture and excess HCl. For TC, TN, and TOC, 30–50 mg of each sample was weighed using an electronic balance (Mettler Toledo, Switzerland) and encapsulated in tin capsules (6 × 6 × 12 mm dimensions). The samples were analyzed using Vario EL Cube (Elementar Analysensysteme GmbH, Germany), where the combustion and reduction temperatures were maintained at 1150 °C and 850 °C, respectively. Mineralized plant-available nitrogen (NO_3_^−^-N and NH_4_^+^-N) was extracted from 2 mm-sieved, air-dried soil using a 2.0 M KCl extraction solution, followed by colorimetric analysis using an AA3 Autoanalyzer (SEAL Analytical, Germany)^34^. Microbial biomass carbon (MBC) was analyzed using the substrate-induced respiration method^35^. Briefly, an aqueous glucose solution was added to a 50 g soil sample, and the moisture was maintained at 50% of the field capacity. The samples were incubated in 1-L canning jars for 3 h at 22 °C, and the amount of carbon dioxide (CO_2_) generated was measured by sampling 0.5 mL of air from the headspace and injecting it into a gas chromatograph (Mandel Scientific Company Inc., ON, Canada). Active carbon, also known as labile carbon or permanganate-oxidizable carbon (POXC) was determined using the protocol by Weil et al.^36^. Air-dried soil (2.5 g) was reacted with 20 mL of 0.02 mol L^− 1^ potassium permanganate (KMnO_4_), where the amount of oxidized carbon is proportional to KMnO_4_. After shaking the samples for 2 min at 120 rpm, a 0.5 mL aliquot was collected and mixed with 49.5 mL of deionized water. Then, the sample absorbance was read with a spectrophotometer at 550 nm (Thermo Electron Corporation, Madison, USA). The activities of β-glucosidase (β-Glc), N-acetyl-β-glucosaminidase (NAG), and acid phosphomonoesterase (Phospo) were analyzed using 4-methylumbelliferyl-linked substrates^37^. A soil suspension was prepared by mixing 1 g of fresh soil with 120 mL of deionized water, and 100 µL of this suspension was dispensed and treated with 50 µL of methyl umbelliferyl substrates in a black 96-well microplate. The microplates were incubated in dark conditions at a temperature of 37 °C for 1 h and analyzed using a fluorescence reader (Twinkle LB 970, Berthold Technologies, Bad Wildbad, Germany) set at 355 nm excitation and 460 nm emission.

### In-season measurements of penetration resistance and soil moisture in the field plots

Soil penetration resistance was measured in June 2023 using a Field Scout SC900 soil compaction meter equipped with a 12.7 mm cone tip (Spectrum Technologies, Inc., Aurora, Illinois, USA) to a depth of 15 cm. At the same time, soil moisture was measured using a TDR 300 Soil Moisture Meter (Spectrum Technologies, Inc., IL, USA). Soil water infiltration was assessed in selected plots (only in the 45 kg N ha^− 1^ subplots) using a mini disk infiltrometer set to a 2 cm suction level. The reason for limiting the measurement to 45 kg N ha^− 1^ subplots that this can represent the impact of different cropping sequences under the given logistical constraints. Infiltration data were recorded at 20-minute intervals over a duration of 2 h as the steady state phase was observed during this period.

### Statistical analysis

All measured variables were analyzed using a linear mixed model approach via the PROC GLIMMIX in SAS OnDemand for Academics (SAS Institute, Inc., Cary, NC). Prior to analysis, the assumption of normality was assessed with PROC UNIVARIATE, while homogeneity of variances was evaluated using Levene’s test and visual inspection of studentized residual plots. As the response variables exhibited Gaussian distribution, an identity link function was applied. Crop sequence, N level, and their interaction were treated as fixed effects. To account for the hierarchical structure of the split-plot design, replications (blocks) and the interaction between replication and cropping sequence (the main-plot error) were considered random effects. The Kenward-Roger’s approximation was used for the denominator degrees of freedom method (ddfm). Analyses for each response variable were constructed in two stages. First alternative variance-covariance structures were evaluated using maximum likelihood estimation. Two candidate structures were compared: (i) the standard split-plot variance structure and (ii) a structure including an additional variance component to accommodate potential heterogeneity among crop sequence treatments. The model selection was based on the Akaike Information Criterion (AIC). After identifying the most appropriate variance structure, the final mixed-model was fitted using restricted maximum likelihood (REML), with the Kenward-Roger method applied to ensure accurate accounting for block-level variability and precise estimation of treatment effects. Type III tests of fixed effects were used to evaluate treatment significance. When significant treatment effects were detected, least squares means were compared using the Tukey procedure at a 5% significance level. Finally, principal component analysis (PCA) was performed using R version 4.4.1 with (The R Foundation) with ‘agricolae’ and ‘PB-Perfect’ packages to assess relationships among soil properties that explained most of the treatment-induced variation.

## Results

### Effect of diversified cropping sequences on soil physical attributes

**Fig. 1:**
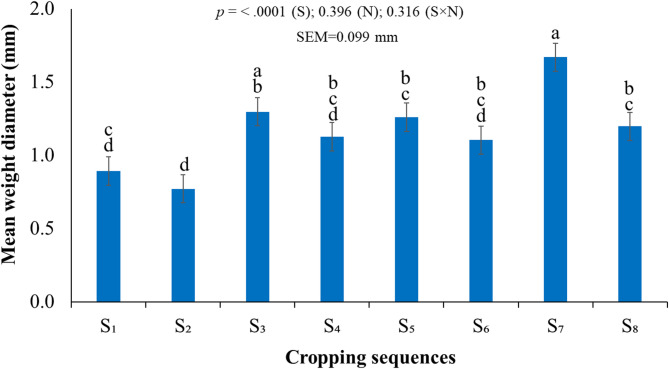
Mean weight diameter of soil aggregates in the 0–15 cm soil depth under eight different cropping systems across three nitrogen fertility levels measured in 2023, after a decade-long experiment, using plots established in 2013 on Luvisolic soil of northwestern Canada. The cropping sequence treatments included S_1_ = Conventional annual rotation; S_2_ = Diversified annual crop sequence; S_3_ = Annual to perennial crop sequence; S_4_ = Legume to vernalizing grass crop sequence; S_5_ = Legume to non-vernalizing grass crop sequence; S_6_ = Legume to turf grass crop sequence; S_7_ = Turf grass to legume crop sequence; and S_8_ = Diversified perennial to annual crop sequence. The Nitrogen rates included 0, 45, and 90 kg N ha^− 1^ applied during the non-legume phase of cropping sequences. Treatment columns with different lowercase letters are significantly different at *p* < 0.05 according to the Tukey procedure. Error bar represents standard error of the mean (SEM) (*n* = 4). S represents *cropping sequences*; N represents *nitrogen levels*, and S×N indicates their interaction.

**Fig. 2:**
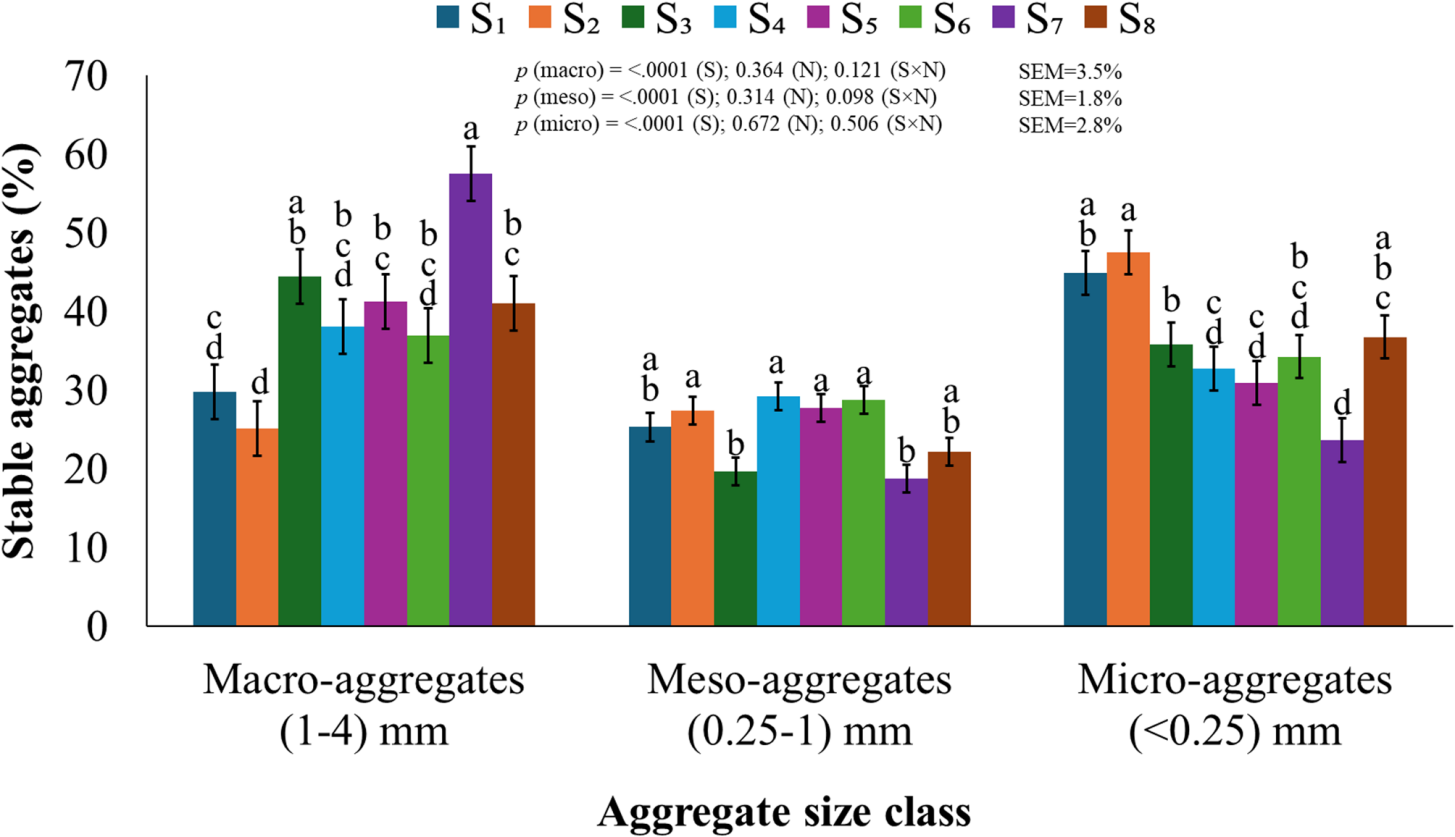
Distribution of soil aggregate size class (%) at 0–15 cm depth under different cropping sequences and nitrogen fertility levels measured in 2023 after ten years, using the experimental plots established in 2013 on Luvisolic soil of northwestern Canada. Aggregate size classes include macro- (1–4 mm), meso- (0.25–1 mm), and micro-aggregates (< 0.25 mm). The cropping sequence treatments included S_1_ = Conventional annual crop sequence; S_2_ = Diversified annual crop sequence; S_3_ = Annual to perennial crop sequence; S_4_ = Legume to vernalizing grass crop sequence; S_5_ = Legume to non-vernalizing grass crop sequence; S_6_ = Legume to turf grass crop sequence; S_7_ = Turf grass to legume crop sequence; and S_8_ = Diversified perennial to annual crop sequence. The Nitrogen rates included 0, 45, and 90 kg N ha^− 1^ applied during the non-legume phase of cropping sequences. Treatment columns with different lowercase letters are significantly different at *p* < 0.05 according to the Tukey procedure. Error bars represent standard error of the mean (SEM) (*n* = 4). S represents *cropping sequences*; N represents *nitrogen levels*; S×N indicates their interaction.

Surface soil (0 to 15 cm depth) physical properties including bulk density, penetration resistance, water content at field capacity, and soil water infiltration rate did not show measurable differences across eight different cropping sequences under three N fertility regimes over a decade (Supplemental Table 1). However, cropping sequences dominated by different crop functional groups exhibited difference in soil aggregate stability. The proportion of macro (1–4 mm) and micro (< 0.25 mm) size groups of water-stable aggregates, as well as the mean weight diameter (MWD) were improved by some perennial-recurring, diversified cropping system treatments (Figs. 1 and 2). The cropping sequence with more recurrent phases of creeping red fescue (S_7_) and the sequence integrating a succession of red clover and meadow bromegrass (S_3_) exhibited 87% and 29% higher MWD, respectively (p *<* 0.0001) compared to conventional annual rotation (S_1_), with no significant effect of N supplementation or sequence-by-N interactions. Similarly, these sequences showed 43% and 57% higher proportion of stable macro-aggregates (p *<* 0.0001), also unaffected by N supplementation or sequence-by-N interactions.

### Soil pH, plant available nitrogen, and total nitrogen in diversified cropping systems

The pH values ranged from 5.7 to 5.9 in the surface soil (0–15 cm) across cropping sequence and did not differ significantly among treatments (*p* < 0.05), indicating that neither long‑term crop diversification nor N fertilization altered soil acidity after 11 years (Table 2).

**Table 2:** Cropping sequences with its key features. Effect of decade-long cropping sequence diversification under different nitrogen fertility levels on soil pH, total nitrogen, and plant available nitrogen at 0–15 cm depth in Dark Gray Luvisolic soil in northwestern Canada. The results are based on assessments conducted in 2023, using the experimental plots established in 2013.

Cropping sequences		Soil pH	Mineral *N* (mg kg^− 1^)		Total *N* (g kg^− 1^)
S. N.	Key features		NO_3_^−^ -*N*	NH_4_^+^-*N*	
S_1_	Conventional annual crop sequence	5.69	26.6ab	7.99	3.5a
S_2_	Diversified annual crop sequence	5.63	25.7ab	11.0	3.2ab
S_3_	Annual to perennial crop sequence	5.55	16.0b	5.32	3.1bc
S_4_	Legume to vernalizing grass crop sequence	5.78	27.4ab	8.85	3.3a-c
S_5_	Legume to non-vernalizing grass crop sequence	5.58	27.5ab	7.83	3.0c
S_6_	Legume to turf grass crop sequence	5.56	30.9a	5.82	3.3a-c
S_7_	Turf grass to legume crop sequence	5.83	18.1ab	6.24	3.6a
S_8_	Diversified perennial to annual crop sequence	5.56	24.8ab	7.31	3.4ab
SEM		0.083	3.182	1.995	0.112
**Nitrogen rates (kg N ha** ^**− 1**^ **)**					
0		5.70	10.2c	4.15b	0.33
45		5.63	26.2b	7.02ab	0.32
90		5.59	34.9a	10.2a	0.33
SEM		0.047	1.984	1.039	0.008
***p-*** **values**					
S		0.100	0.012	0.541	< 0.0001
N		0.100	< 0.0001	0.002	0.474
S×N		0.988	0.021	0.426	0.993

The nitrogen rates included 0, 45, and 90 kg N ha^− 1^ applied during the non-legume phase of cropping sequences. Numbers followed by different letters are significantly different at *p* < 0.05 according to Tukey’s test. SEM stands for standard error of the mean; S represents cropping sequences; N represents nitrogen; S×N indicates their interaction.

In contrast, the results revealed differences in NO₃⁻-N and total N due to legacies of cropping sequences and N fertilization levels over the production years (Table 2). Across cropping sequences, NO₃⁻-N ranged from 16.0 mg kg⁻¹ in S_3_ to 30.9 mg kg⁻¹ in S_6_, reflecting the influence of crop sequence composition. Nitrogen application rates had a strong effect on NO₃⁻-N (*p* < 0.0001) with concentrations increasing from 10.2 mg kg⁻¹ at 0 N to 34.9 mg kg⁻¹ at 90 kg N ha⁻¹. These results indicate that NO₃⁻-N dynamics were driven primarily by fertilizer inputs rather than direct contributions from crop functional traits. Similarly, significant differences in total soil N content across cropping sequences are also not logically attributable to crop functional groups. The significant interaction between cropping sequence and N rates further indicates that the magnitude of the N-rate response varied among sequences.

### Soil carbon pools

In the surface soil (0–15 cm), the response of carbon fractions varied significantly (*p* < 0.05) with cropping sequences after a decade-long crop diversification (2023), regardless of N fertilization treatments (Table 3). We found no significant interaction between the cropping sequence and N fertilizer rate for soil C (*p* > 0.05, Table 3). The cropping sequence integrating creeping red fescues (S7 and S8) consistently exhibited higher SOC and TC content, although most sequences were statistically similar.

**Table 3:** Cropping sequences with its key features. Effect of decade-long cropping sequence diversification under different nitrogen fertility levels on soil total (TC) and organic C (SOC), permanganate oxidizable C (POXC), and microbial biomass C (MBC) at 0–15 cm depth in Dark Gray Luvisolic soil in northwestern Canada. The results are based on assessments conducted in 2023, using the experimental plots established in 2013.

Cropping sequences		SOC (g kg^− 1^)	POXC (mg kg^− 1^)	MBC (mg kg^− 1^)	Total C (g kg^− 1^)
S. N.	Key features				
S_1_	Conventional annual crop sequence	28.8a	725ab	676ab	31.2a
S_2_	Diversified annual crop sequence	27.1ab	728ab	498 cd	28.6ab
S_3_	Annual to perennial crop sequence	25.8ab	671b	526 cd	27.6ab
S_4_	Legume to vernalizing grass crop sequence	26.5ab	716ab	543bc	27.9ab
S_5_	Legume to non-vernalizing grass crop sequence	24.5b	672b	392de	25.9b
S_6_	Legume to turf grass crop sequence	27.5ab	801a	384e	29.6ab
S_7_	Turf grass to legume crop sequence	29.0a	817a	730a	31.4a
S_8_	Diversified perennial to annual crop sequence	29.3a	805a	702a	30.7a
SEM		1.124	40.76	49.47	1.067
***p-*** **values**					
S		0.001	0.003	< 0.001	0.001
N		0.572	0.238	0.111	0.279
S×N		0.976	0.995	0.045	0.996

The nitrogen rates included 0, 45, and 90 kg N ha^− 1^ applied at the non-legume phase of cropping sequences. Numbers followed by different letters are significantly different at *p* < 0.05 according to Tukey’s test. SEM stands for standard error of the mean; S represents cropping sequences, N represents nitrogen; S×N indicates their interaction.

Significant differences were observed in POXC and MBC contents among the cropping sequences, which evolved over a decade from 2013 to 2023 (Table 3). Similar to SOC, POXC content was approximately 10–12% higher in fescue-integrated cropping sequences (S_6_, S_7_ and S_8_), although these values were statistically similar to those in meadow bromegrass-included sequence as well as annual cropping sequences (S_1_ and S_2_) (Table 3). Two of the fescue-integrated sequences showed 3–7% higher MBC than most cropping sequences, while staying on par with wheat-canola alternating sequence.

### Soil extracellular enzyme activities

β-glucosidase and β-N-acetyl-glucosaminidase, key enzymes catalyzing C- and N-cycling respectively, were influenced by cropping sequences, with no significant effect of N fertility levels or sequence-by-N interactions. Consistent with other soil functionality parameters, two creeping red fescue-integrated sequences (S_7_ and S_8_) showed higher β-glucosidase acclivity, though the values were statistically similar to meadow-bromegrass-included (S_4_) and conventional wheat-canola alternating sequences (S_1_) after a decade of crop diversification (*p* < 0.001, Table 4). These sequences also showed similar performance for β-N-acetyl-glucosaminidase activity, remaining on par with two perennial legume-integrated sequences (S_4_ and S_6_) and the annual diversified sequence (S_2_). The activity of acid phosphomonoesterase, an enzyme that catalyzes the mineralization of organic phosphorus, showed no response to long-term crop diversification or fertility regimes.

**Table 4:** Cropping sequences with its key features. Soil extracellular enzyme activities at 0–15 cm soil depth across different cropping sequences under three nitrogen levels in Dark Gray Luvisolic soil of northwestern Canada. The results are based on assessments conducted in 2023, using the experimental plots established in 2013.

Cropping sequences		β-glucosidase	β-*N*-acetyl-glucosaminidase	Acid phosphomonoesterase
S. n.	Key features	(pmol MUB g^− 1^ h^− 1^)		
S_1_	Conventional annual crop sequence	1577a-d	436ab	3931
S_2_	Diversified annual crop sequence	1508b-d	385ab	3783
S_3_	Annual to perennial crop sequence	1492b-d	342b	3687
S_4_	Legume to vernalizing grass crop sequence	1648a-c	394ab	3453
S_5_	Legume to non-vernalizing grass crop sequence	1183d	308b	3139
S_6_	Legume to turf grass crop sequence	1416 cd	377ab	3563
S_7_	Turf grass to legume crop sequence	1950ab	385ab	3619
S_8_	Diversified perennial to annual crop sequence	2030a	522a	3826
SEM		132.70	41.27	268.53
***p-*** **values**				
S		< 0.0001	0.004	0.094
N		0.468	0.947	0.092
S×N		0.707	0.288	0.707

The nitrogen rates included 0, 45, and 90 kg N ha^− 1^ applied at the non-legume phase of cropping sequences. Numbers followed by different letters are significantly different at *p* < 0.05 according to Tukey’s test. SEM stands for standard error of the mean; S represents cropping sequences, N represents nitrogen; S*N indicates their interaction.

### Relationship among soil properties influenced by cropping sequences

**Fig. 3:**
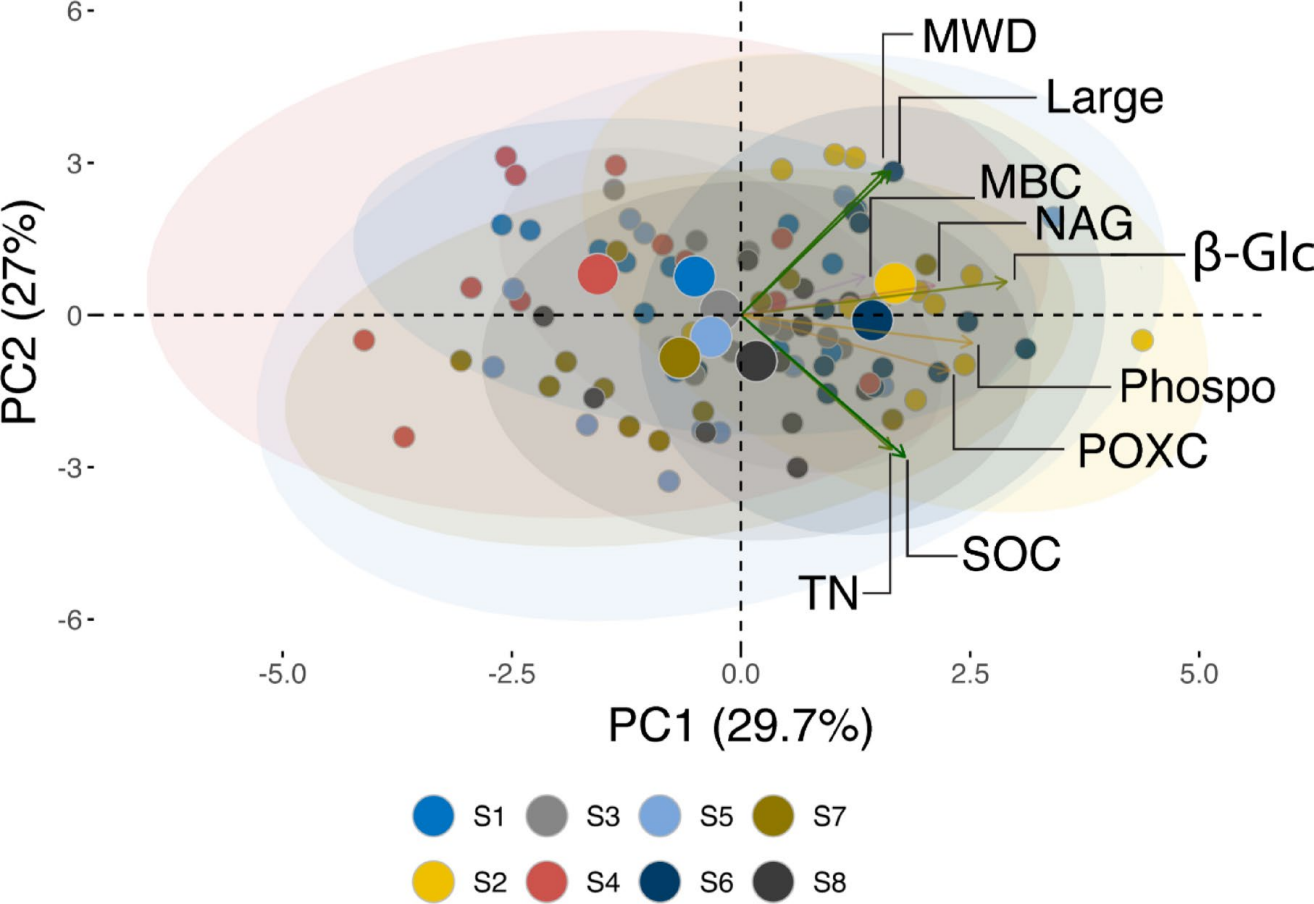
Principal component analysis (PCA) biplot of soil properties across eight cropping sequences (S_1_ – S_8_) based on soil samples collected in 2023 from the long-term experiment established in 2013. The cropping sequence treatments included S_1_ = Conventional annual crop sequence; S_2_ = Diversified annual crop sequence; S_3_ = Annual to perennial crop sequence; S_4_ = Legume to vernalizing grass crop sequence; S_5_ = Legume to non-vernalizing grass crop sequence; S_6_ = Legume to turf grass crop sequence; S_7_ = Turf grass to legume crop sequence; and S_8_ = Diversified perennial to annual crop sequence. Large circles represent group centroids for each cropping sequence, while small dots indicate individual observations. MWD, mean weight diameter (mm); Large, macro-aggregates (4.0–1.0 mm); MBC, microbial biomass carbon (mg kg⁻¹); NAG, β-N-acetyl-glucosaminidase (pmol MUB g⁻¹ h⁻¹); β-Glc, β-glucosidase (pmol MUB g⁻¹ h⁻¹); Phospo, acid phosphomonoesterase (pmol MUB g⁻¹ h⁻¹); POXC, permanganate oxidizable carbon (mg kg⁻¹); SOC, soil organic carbon (%) and. TN, total nitrogen (%).

Principal component analysis (PCA) revealed two principal components (PC1 and PC2) accounted for 56.7% of the total variance, with PC1 explaining 29.7% and PC2 explaining 27.0% (Fig. 3). The biplot reveals distinct loading patterns for the measured variables. All soil variables included in the analysis showed positive loadings on PC1. Soil structural indicators MWD and macroaggregates (Large) were strongly correlated with each other (*r* = 0.99***; *p* < 0.001), and these variables along with MBC, NAG and β-Glc loaded onto the positive direction of both PC1 and PC2 (Supplementary Section B), suggesting a coordinated increase with both soil biological activities and structural stability. Acid phosphomonoesterase (Phospho) showed positive loadings on PC1. Soil organic carbon (SOC) and TN exhibited a strong positive correlation (*r* = 0.92; p = < 0.001), as indicated by their overlapping vectors. These variables, along with POXC, were primarily associated with the positive direction of PC1. These suggest that the variables governing SOC, structural stability, soil microbial activities, and nutrient cycling serve the soil processes in coordinated fashion (Fig. 3; Supplementary Section B).

The distribution of cropping sequences across the PCA showed partial separation among treatments. Notably the clusters of diversified annual cropping sequence (S_2_) and cropping sequence with intermittent inclusion of creeping red fescue and red clover (S_6_) exhibited high positive scores along PC1. The sequences that intermittently included alsike or red clover with the annual crops and/or perennial timothy grass (S_3_ and S_5_) and the sequence that intermittently included creeping red fescue and alsike clover (S_8_) with the annual crops showed significant overlap near origin, indicating a high degree of similarity in their soil property profiles. The annual wheat-canola alternation sequence (S_1_) and creeping red fescue repetition with short intervention by legumes (S_7_) did not show a negative association with PC1, which in turn is positively associated with all soil quality variables. It is noteworthy that the S_3_ which had continuous canola for four consecutive years from 2013 to 2016 followed by perennial meadow bromegrass leading to annual crops till 2023, is also positioned near the center of the biplot, indicating a soil property profile similar to several other sequences (S_1_, S_5_, S_7_, and S_8_). In contrast, S_4_ is clearly separated and lies on the negative side of PC1. However, PC1 describes a gradient in which soil structural stability, biological activity, and carbon and nitrogen pools increase concurrently. Overall, the PCA results show limited differentiation among most of the diversified cropping systems evaluated in this study.

## Discussion

### No influence of cropping sequences on major soil physical properties

Our decade-long study on Luvisolic soil in northwestern Alberta, Canada revealed that cropping sequence diversification exerted no measurable influence on major soil physical properties such as bulk density, penetration resistance (an indicator of soil compaction), water infiltration, water holding capacity, regardless of N fertility regimes. Bulk density values of surface soil layer (0–15 cm depth) across the cropping sequences remained around 1.5 g cm^− 3^, indicating a marked increase from the corresponding baseline value of 1.3 g cm^− 3^ recorded in 2013. This increase is likely attributable to the continuous use of heavy machineries for agronomic operations and soil sampling process. Although the bulk density values appear within the typical range of clay loam soils^38^, negative effects of increased bulk density have manifested as staggered or poorer emergence of crops in more recent years. No influence of cropping sequences in this study aligns with a previous study on a Webster clay loam soil in Minnesota, where no significant changes in bulk density occurred after 14 years under continuous corn, continuous soybean, and alternating corn-soybean cropping systems^39^. Soil bulk density also serves as an indicator of soil penetration resistance and dynamically influences various soil properties that impact soil functionality and productivity^40^, which is also reflected in the present study with no measurable impacts on the soil penetration resistance across different cropping sequences. Similar water retention and infiltration patterns observed across cropping sequences reflected the interrelationship among soil physical properties. Though this study did not include a heavy farm machinery factor on soil compaction, it is reasonable to argue that findings suggest that crop management factors such as use of heavy machineries may mask the potential benefits of crop diversification in improving some physical properties of Luvisolic soils which predominate in the Peace region of Canada.

### Perennial integration enhances soil aggregate stability

Despite the absence of measurable effects on major physical properties, significant improvements were observed in soil aggregate stability under the perennial integrated sequences. In particular, the creeping red fescue phase (S_7_) with highest MWD and greater proportion of water-stable macro-aggregates contributed in improving soil aggregate stability. The meadow bromegrass based sequence (S_3_) also contributed in improving soil aggregate stability, although it did not differ significantly from other diversified perennial sequences (S_4_, S_5_, S_6_, or S_8_). Overall, the inclusion of perennial grasses collectively supported enhanced soil structural resilience relative to the annual sequences (S_1_ and S_2_), as reflected in higher soil aggregate MWD and a greater proportion of water‑stable macro‑aggregates. These findings are supported by previous research, which reported that perennial grasses contribute to stable aggregate formation compared to their annual counterparts due to higher fine root biomass and greater root length density in the surface soil layer^10,41,42^. Reportedly, the fescue rhizosphere with a fibrous and extensive root network can physically intertwine soil particles to promote soil aggregation^43^. In a previous study, higher SOC, mobile humic substances, and mobile humic acids accumulation were observed in surface soil after five years^44^. These accumulated C inputs into the microbial community lead to greater extracellular polysaccharide production, also known as transient binding agents in soil that enhance aggregate stabilization^45–47^. Similarly, the sequences that included alsike clover at an earlier phase (S_5 and_ S_8_) showed significantly greater aggregate stability compared to S_2_. The soil-transferred legacy effects of legumes on the following crops is previously documented^48^. Therefore, the inclusion of alsike clover as preceding crops might have contributed to enhance aggregate stability when combined with perennial grasses (as in S_5_ and S_8_). In contrast, the lower MWD in the conventional annual rotation (S_1_) and diversified annual rotation (S_2_) is consistent with previous findings that annual cropping practices can lead to weaker aggregate stability and lower MWD^49–51^. In the current study, N fertilization did not significantly affect aggregate stability, highlighting the dominant role of crop functional diversity over N input.

### No specific trend of soil pH and soil nitrogen under different cropping sequences

Soil pH as a major soil chemical property remained stable across the cropping sequences, irrespective of N fertility regimes. The SOC content, ranging from 26 to 31 g C kg^− 1^ across the cropping sequences in this study, likely provided similar buffering capacity against pH variation. Soils with higher OM content generally exhibit greater buffering capacity against pH fluctuations^52^. Additionally, the assimilation of N as NH₄⁺ or N₂ into legume nodules leads to H⁺ ion release into the rhizosphere, while plant uptake of N as NO₃⁻ by cereals and grain crops results in OH⁻ production^53,54^, thereby balancing pH changes that might have occurred in our cropping system treatments.

The soil N dynamics were more strongly influenced by N fertilization than by cropping sequence legacies. The pronounced increase in NO₃⁻-N with higher N application rates, and the significant interaction effects indicate that fertilizer inputs were the primary driver of inorganic N at post-harvest. These results contradict with most studies, which report increased N accrual under diversified, legume-integrated cropping systems. Possibly significant amount of non-symbiotic N fixation^55,56^ and drought stress^57^ which prevailed at varying severity levels since 2021 masked the legacy effects of diversified, legumes integrated cropping sequences. Conversely, the lowest NO₃⁻-N levels in annual to perennial crop sequence (S_3_) at post-harvest indicates that the applied N was used more efficiently by the perennial grass seed crops. The deeper and more extensive root systems and longer growing periods allowed them better acquisition and assimilation of applied N instead of leaving it in the soil. The reduction of residual NO₃⁻-N has important ecological implications as it reduces the risk of NO₃⁻ leaching and improves synchronization between N supply and plant uptake. Thus, the inclusion of perennial grass seed crops can promote efficient nutrient cycling.

### Soil carbon pool transformations in response to cropping sequence diversity

Carbon pools responded positively to perennial integration, although the magnitude of this response varied among sequences. Sequences containing creeping red fescue (S_7_, S_8_) showed higher SOC, TC, and labile carbon fractions (POXC) values primarily in comparison to S_5_. Microbial biomass C also increased under these sequences, reinforcing the role of perennial phases in promoting microbial activity and carbon sequestration. However, SOC in S_7_ and S_8_ was not significantly different from SOC in the annual sequences (S_1_ and S_2_) indicating that the present study does not provide consistent evidence that perennial cropping systems enhance SOC relative to annual systems. Nitrogen fertilization had no measurable long-term effects on these parameters. The significant response of SOC fractions to cropping sequence irrespective of N fertilization rates clearly highlights the dominant role of crop sequence treatment in regulating soil C dynamics. These results are consistent with previous findings^58–60^ demonstrating the positive impact of perennial forage incorporation on SOC accumulation over time, while also highlighting that such effects are not always statistically distinguishable from those of annual systems over the same timeframe. The increase in SOC with the recurrence of fescue in the cropping sequence may be linked to continuous living root activity, steady turn-over of dead roots and aboveground residues, and less soil disturbance during the perennial phase^61–63^. Usually, the decomposability of creeping red fescue thatch (a layer of living and dead plant tissues derived from crowns, stems, and roots) is very slow due to high lignin content, which is also highly recalcitrant to microbial breakdown^64,65^. However, the fescue dominant system was better suited for enhancing and stabilizing SOC over the long term due to its dense root systems, slower root decomposition, and continuous stimulation of microbial activity^59,66^. These results indicate that appreciable changes in SOC require a long-term recurrence of perennial crops, and the benefits may be most evident when compared with sequences that inherently accumulate less carbon.

Similar to SOC, the elevated levels of POXC and MBC in sequences dominated by fescue and alsike clover suggest that the long-term presence of perennial crops enhances belowground biomass production, promotes continuous rhizodeposition, and stimulates microbial activity^58–60,66^. Usually, POXC is more stable than MBC. Therefore, the greater variability observed in MBC compared to POXC likely reflects the cropping systems differences in length of perennial phases, legumes, residue quality, and microenvironmental conditions. The increased accumulation and turnover of biologically active carbon under these systems can improve soil biological function and nutrient cycling. In contrast, lower TC may be associated with reduced biomass production of alsike clover in the initial phase^26^ or due to the positive priming effect of low C: N leguminous residues (that accelerates the breakdown of older and more stable organic matter)^67,68^.

### Soil extracellular enzyme activities supporting soil functionality

Despite significant differences in the β-glucosidase and β-N-acetyl-glucosaminidase activities across cropping sequences, no logical patterns attributable to crop diversification levels are discernible. These results are not in agreement with previous studies reporting that these enzyme activities increase with the duration of legume phases in rotations^69,70^. The detritus of legume roots also stimulates enzyme activities in soil^71^, which is primarily driven by the release of organic substrates that serve as energy sources for soil microorganisms. Furthermore, microbes secrete extracellular enzymes to break down complex organic compounds into simpler forms and contribute to the formation of SOM, nutrient cycling, and soil fertility improvement^17,71–73^.

The activity of acid phosphomonoesterase enzymes, which catalyze the mineralization of organic P, remained unaffected, suggesting limited influence of crop diversification on phosphorus mineralization. Or results do not align with a previous study that reported an increase in acid phosphatase enzyme activity with the incorporation of legumes into crop rotations^74^. The enzyme activity is considered to be higher in acidic soil^75^ similar to our research site. Moreover, the phosphomonoesterase enzyme secretion depends on plant types^74,76–78^, soil microbial activity^75,79,80^ and phosphorus availability^81–83^. As discussed before, heavy machinery-based agronomic management, and high frequency and/or intensity of stress during growing seasons may have attenuated the results.

### Soil property interactions under diverse cropping systems

The PCA analysis revealed a strong multivariate structure in soil properties. Although the first principal component (PC1) explained 29.7% of the total variation governing the soil quality parameters, it represented an integrated soil quality axis combining organic matter pools, structural stability, and microbial activity. The positive association among these indicators suggests that improvements in one parameter such as organic matter content or microbial activity reinforces improvement in other quality parameters, creating synergistic effects on soil functioning^84^. The coordinated responses of soil quality parameters reflect the underlying soil ecological processes such as residue decomposition and aggregate formation, which link soil biological activity with physical stability and nutrient cycling^71–73^.

The limited separation of cropping sequences in PCA space also indicates similar effects of rotational design with diverse crop functional traits on soil properties. Although some sequences containing perennial phases or legumes aligned with slightly higher scores along PC1, these features did not consistently result in higher soil quality scores across all systems. For example, cropping sequences with an initial red clover phase and annual sequences with extended canola phases occupied positions associated with lower values of soil quality indicators, particularly those related to carbon pools, microbial activity, and aggregate stability. However, the variation observed among a few sequences suggests that specific rotational designs may still influence soil properties related to carbon inputs, microbial activity, and aggregation. Similar trends have been reported in previous studies, where soil biological and biochemical properties were found to be significantly influenced by diversified cropping systems^69,85–87^. Overall, the PCA results reinforce the concept multidimensional property of soil and support the hypothesis that diversified cropping positively influences key soil attributes including carbon dynamics, microbial activity, and aggregate stability.

## Conclusion

This study, based on decade-long cropping sequence experimental plots under no-till management, demonstrated that crop diversification exerted a facultative impact on soil properties, whereas N fertilization had no effect. Although crop diversification did not influence soil bulk density, penetration resistance, water infiltration, and pH, the integration of some perennial forage seed crops including creeping red fescue, meadow brome grass, red clover and alsike clover into annual cropping systems improved soil aggregate stability. This improvement was most evident in sequences involving balanced alternation of these perennial grasses and forage legume seed crops, underscoring the role of crop functional diversity over nitrogen fertilization in enhancing the capacity of soil to maintain functional and structural integrity. The fescue, meadow brome and clover-integrated sequences were also positively associated with long-term accrual of soil organic carbon and nitrogen, increased labile (active) carbon fractions and microbial biomass carbon, and enhanced level of carbon- and nitrogen-cycling enzyme activities. These findings highlight the importance of strategic inclusion of perennial crops for improving soil biological functioning, carbon sequestration, and structural stability, which eventually can contribute to the improvement of soil physical properties and create a more conducive environment for crop performance. It is suspected that heavy machinery-based agricultural operations under no-till management masked the subtle beneficial effects of crop diversification on soil physical properties. Therefore, further studies should consider strategic interventions such as occasional tillage and cover cropping to assess whether these practices can alleviate soil compaction resulting from heavy machinery operations and trigger cascading improvement in soil physical properties.

## Supplementary Information

Below is the link to the electronic supplementary material.

Supplementary Material 1

## Data Availability

The datasets generated during and/or analysed during the current study are available from the corresponding author on reasonable request.
