# Supplementary material for: Perennial and annual cropping sequences differentially influence soil functionality in Luvisolic soils
Source: Sci Rep. 2026 Aug 18;16:25889. doi: 10.1038/s41598-026-59597-2 (PMC13486670; doi:10.1038/s41598-026-59597-2)
Supplement: Supplementary file 1 [file 41598_2026_59597_MOESM1_ESM.docx]

**Supplemental Material Section**

**Supplementary Section A: Results of soil physical Properties**

**Supplementary Table 1.** Effects of cropping sequences on soil physical properties (0-15 cm depth) under variable nitrogen inputs in a Dark Gray Luvisolic soil of northwestern Alberta.

| Cropping sequences | | Bulk density  (g cm^-3^) | Soil resistance  (PSI) | Water holding at field capacity  (%) | Water infiltration rate  (mm h^-1^)^♠^ |
| --- | --- | --- | --- | --- | --- |
| S.N. | **Key features** |  |  |  |  |
| S_1_ | Conventional annual crop sequences | 1.50 | 107 | 45.0 | 24.6 |
| S_2_ | Diversified annual crop sequences | 1.47 | 116 | 44.9 | 26.0 |
| S_3_ | Annual to perennial crop sequences | 1.46 | 114 | 44.6 | 12.4 |
| S_4_ | Legume to vernalizing grass crop sequences | 1.51 | 105 | 41.5 | 25.3 |
| S_5_ | Legume to non-vernalizing grass crop sequences | 1.49 | 106 | 41.7 | 14.8 |
| S_6_ | Legume to turf grass crop sequences | 1.49 | 100 | 45.9 | 26.3 |
| S_7_ | Turf grass to legume crop sequences | 1.50 | 102 | 41.4 | 14.6 |
| S_8_ | Diversified perennial to annual crop sequences | 1.44 | 122 | 44.5 | 21.8 |
| *p-values* | |  |  |  |  |
| S | | 0.321 | 0.291 | 0.125 | 0.075 |
| N | | 0.710 | 0.322 | 0.187 | - |
| S*N | | 0.432 | 0.979 | 0.370 | - |

The cropping sequence treatments included S_1_ = Conventional annual rotation; S_2_ = Diversified annual rotation; S_3_ = Annual to perennial rotation; S_4_ = Legume to vernalizing grass; S_5_ = Legume to non-vernalizing grass; S_6_ = Legume to turf grass; S_7_ = Turf grass to legume; and S_8_ = Diversified perennial to annual. The nitrogen rates included 0, 45, and 90 kg N ha^-1^ applied at the non-legume phase of each cropping sequences. ♠ = Infiltration was measured in each of the tested sequences fertilized with 45 kg N ha^-1^, and the data were analyzed using one-way ANOVA. Numbers followed by different letters are significantly different at p < 0.05 according to Tukey’s test.

**Supplementary Section B: Results of principal component analysis (PCA)**

1. **Correlations**

|  | **POXC** | **MBC** | **TN** | **SOC** | **MWD** | **Large** | **β-Glc** | **NAG** | **Phospho** |
| --- | --- | --- | --- | --- | --- | --- | --- | --- | --- |
| **POXC** | 1.000 | -0.025 | 0.396*** | 0.410*** | 0.085 | 0.085 | 0.312** | 0.260* | 0.354*** |
| **MBC** | -0.025 | 1.000 | 0.052 | 0.038 | 0.140 | 0.185 | 0.380*** | 0.283** | 0.022 |
| **TN** | 0.396*** | 0.052 | 1.000 | 0.920*** | -0.149 | -0.136 | 0.106 | -0.056 | 0.174 |
| **SOC** | 0.410*** | 0.038 | 0.920*** | 1.000 | -0.207* | -0.194 | 0.143 | 0.036 | 0.277** |
| **MWD** | 0.085 | 0.140 | -0.149 | -0.207* | 1.000 | 0.992*** | 0.300** | 0.147 | 0.031 |
| **Large** | 0.085 | 0.185 | -0.136 | -0.194 | 0.992*** | 1.000 | 0.319** | 0.160 | 0.034 |
| **β-Glc** | 0.312** | 0.380*** | 0.106 | 0.143 | 0.300** | 0.319** | 1.000 | 0.459*** | 0.589*** |
| **NAG** | 0.260* | 0.283** | -0.056 | 0.036 | 0.147 | 0.160 | 0.459*** | 1.000 | 0.457*** |
| **Phospho** | 0.354*** | 0.022 | 0.174 | 0.277** | 0.031 | 0.034 | 0.589*** | 0.457*** | 1.000 |
| Signif. codes: 0 ‘***’ 0.001 ‘**’ 0.01 ‘*’ 0.05 ‘.’ 0.1 ‘ ’ 1 | | | | | | | | | |
| Source: PCA - Data analysis | | | | | | | | | |

| 2. **2. Eigenvalues and Eigenvectors** | | | | | | | | | |
| --- | --- | --- | --- | --- | --- | --- | --- | --- | --- |
| Package used : agricolae; PB-Perfect | | | | | | | | | |
|  | PC1 | PC2 | PC3 | PC4 | PC5 | PC6 | PC7 | PC8 | PC9 |
| **Eigen values and Variance %** | | | | | | | | | |
| **Eigen Value** | 2.81 | 2.40 | 1.33 | 1.03 | 0.55 | 0.53 | 0.29 | 0.05 | 0.01 |
| **Variance %** | 31.24 | 26.67 | 14.75 | 11.50 | 6.10 | 5.90 | 3.19 | 0.60 | 0.08 |
| **Cumulative Variance %** | 31.24 | 57.90 | 72.65 | 84.15 | 90.24 | 96.14 | 99.33 | 99.92 | 100.00 |
| **Eigenvectors** | | | | | | | | | |
| **POXC** | 0.33 | -0.25 | 0.03 | 0.41 | 0.78 | -0.15 | 0.10 | -0.05 | 0.02 |
| **MBC** | 0.24 | 0.12 | -0.05 | -0.82 | 0.34 | 0.04 | 0.36 | -0.01 | -0.03 |
| **TN** | 0.16 | -0.52 | 0.41 | -0.15 | -0.13 | -0.10 | -0.06 | 0.70 | -0.03 |
| **SOC** | 0.17 | -0.55 | 0.30 | -0.14 | -0.25 | -0.07 | 0.02 | -0.71 | 0.02 |
| **MWD** | 0.35 | 0.40 | 0.42 | 0.15 | -0.12 | -0.05 | 0.08 | -0.05 | -0.70 |
| **β-Glc** | 0.49 | 0.01 | -0.18 | -0.11 | 0.04 | 0.47 | -0.70 | -0.01 | -0.01 |
| **NAG** | 0.37 | 0.04 | -0.45 | -0.06 | -0.23 | -0.76 | -0.14 | 0.03 | 0.00 |
| **Phospho** | 0.39 | -0.18 | -0.39 | 0.24 | -0.33 | 0.39 | 0.58 | 0.11 | 0.00 |
| **Large** | 0.36 | 0.40 | 0.42 | 0.10 | -0.12 | -0.04 | 0.09 | 0.00 | 0.71 |
|  | | | | | | | | | |

| 3. **PCA - Trait statistics** | | | | | |
| --- | --- | --- | --- | --- | --- |
| Package used : agricolae; PB-Perfect | | | | | |
|  | PC1 | PC2 | PC3 | PC4 | PC5 |
| **Correlation between PCs and Traits** | | | | | |
| **POXC** | 0.61 | -0.29 | 0.16 | -0.33 | 0.45 |
| **MBC** | 0.36 | 0.20 | -0.21 | 0.84 | 0.08 |
| **TN** | 0.44 | -0.71 | 0.44 | 0.20 | -0.05 |
| **SOC** | 0.48 | -0.75 | 0.32 | 0.15 | -0.05 |
| **MWD** | 0.42 | 0.76 | 0.47 | -0.07 | -0.01 |
| **β-Glc** | 0.78 | 0.18 | -0.27 | 0.04 | -0.34 |
| **NAG** | 0.57 | 0.16 | -0.53 | -0.02 | 0.44 |
| **Phospho** | 0.68 | -0.15 | -0.39 | -0.36 | -0.35 |
| **Large** | 0.44 | 0.76 | 0.47 | -0.03 | -0.01 |
| **Quality of representation of Traits on PCs** | | | | | |
| **POXC1** | 0.37 | 0.09 | 0.03 | 0.11 | 0.21 |
| **MBC1** | 0.13 | 0.04 | 0.04 | 0.70 | 0.01 |
| **TN1** | 0.19 | 0.50 | 0.19 | 0.04 | 0.00 |
| **SOC1** | 0.23 | 0.56 | 0.10 | 0.02 | 0.00 |
| **MWD1** | 0.17 | 0.58 | 0.23 | 0.00 | 0.00 |
| **β-Glc 1** | 0.60 | 0.03 | 0.07 | 0.00 | 0.11 |
| **NAG1** | 0.32 | 0.02 | 0.28 | 0.00 | 0.20 |
| **Phospho1** | 0.46 | 0.02 | 0.16 | 0.13 | 0.12 |
| **Large1** | 0.19 | 0.58 | 0.22 | 0.00 | 0.00 |
| **Contributions (%) of the Traits to the Principal Components** | | | | | |
| **POXC2** | 13.92 | 3.56 | 2.00 | 10.96 | 31.65 |
| **MBC2** | 4.90 | 1.69 | 3.27 | 69.42 | 0.89 |
| **TN2** | 7.24 | 20.73 | 14.73 | 3.96 | 0.38 |
| **SOC2** | 8.58 | 23.12 | 7.89 | 2.14 | 0.45 |
| **MWD2** | 6.55 | 23.92 | 17.12 | 0.47 | 0.03 |
| **β-Glc2** | 22.57 | 1.28 | 5.53 | 0.19 | 17.37 |
| **NAG2** | 11.99 | 1.01 | 21.14 | 0.02 | 30.43 |
| **Phospho2** | 17.08 | 0.90 | 11.80 | 12.76 | 18.78 |
| **Large2** | 7.16 | 23.78 | 16.50 | 0.08 | 0.03 |
|  | | | | | |

4. Contributions of Variables to two major principal components
